# CircRNA-NOLC1 mediates Insulin-like growth factor 1 receptor via performing as a ceRNA of miRNA-140–5p to facilitate testicular germ cell tumor advancement

**DOI:** 10.1016/j.clinsp.2025.100629

**Published:** 2025-05-13

**Authors:** Feng Lin, Xianming Yao, ShuoShuo Zhang, Huajun Yang

**Affiliations:** a Department of Urology, Hangzhou Children's Hospital, Hangzhou City, Zhejiang Province, PR China; b Department of Urology, Heji Hospital of Changzhi Medical College, Changzhi City, Shanxi Province, PR China

**Keywords:** CircNOLC1, Testicular germ cell tumor, MicroRNA-140–5p, Insulin-like growth factor receptor

## Abstract

**Objective:**

The study explored the molecular mechanism of circNOLC1 influencing Testicular Germ Cell Tumor (TGCT) progression.

**Methods:**

The study used TGCT tissue samples and cell lines for investigations. The circNOLC1 and miR-140–5p expression in TGCT tissues were done through RT-qPCR. Analysis of the association of circNOLC1 with TGCT clinic-pathological features was also done. Transfections of circNOLC1, miR-140–5p or Insulin-like GrowthFfactor 1 Receptor (IGF1R)-related sequences or plasmids into TCAM-2 and NCCIT TGCT cells were done. The circNOLC1, miR-140–5p, and IGF1R mRNA expression in cells were done through RT-qPCR. Interaction between miR-140–5p, circNOLC1, and IGF1R was examined using a dual-luciferase reporter assay. MTT assay and colony-forming assay were used to investigate cell proliferation. Apoptosis was determined by flow cytometry. Transwell assay was used to investigate cell invasion. IGF1R protein expression was determined through a western blot.

**Results:**

Increased circNOLC1 in TGCT tissues was correlated with lymph node metastasis, clinical stage, and pathological grade of TGCT patients. CircNOLC1 knock-down inhibited TGCT cell proliferation, colony formation, and invasion, and promotedapoptosis. MiR-140–5p was reduced while IGF1R was upregulated in TGCT tissues and cell lines. Moreover, miR-140–5p mimic could reverse the effect of circNOLC1 knock-down on malignant behaviors of TGCT cells. The authors demonstrated that elevated IGF1R reversed the negative effect of miR-140–5p mimic on TGCT cell proliferation, colony formation, and invasion. CircNOLC1 can act as a sponge of miR-140–5p to up-regulate the IGF1R expression level.

**Conclusion:**

The study highlights that circNOLC1 promotes the progression of TGCT by regulating the miR-140–5p/IGF1R axis.

## Highlights

• CircNOLC1 and IGF1R are upregulated while miR-140–5p is downregulated in TGCT.
• CircNOLC1 can act as a sponge of miR-140–5p to up-regulate IGF1R.
• Silencing circNOLC1 or overexpressing miR-140–5p inhibits TGCT cell proliferation, colony formation, and invasion, and promotes cell apoptosis.
• CircNOLC1 promotes the progression of TGCT by modulating the miR-140–5p/IGF1R axis.

## Introduction

Testicular cancer is a prevalent malignant tumor in adult males aged 20 to 35, among which Testicular Germ Cell Tumor (TGCT) takes up 90 %∼95 %. Meanwhile, germ cell tumors are subdivided into Seminoma (SEM) and Non-Seminoma (NSEM), with SEM being the most prevalent.^1^^,^^2^ Computed tomography, scrotal ultrasound, and clinical examination are critical TGCT diagnostic means. Additionally, serum tumor markers such as lactate dehydrogenase, b-human chorionic gonadotrophin, and α fetoprotein are crucial markers for testicular cancer diagnosis. These markers are also crucial for the prognosis, diagnosis, histology, and typing of TGCT.^3^ However, the test of these serum tumor markers frequently leads to false positive or negative results of the tumor types and comorbidities.^4^ Consequently, research on developing new tumor markers with high specificity and sensitivity are ongoing.^5^ Although TGCT has a high recovery rate, poor prognosis and increased deaths are still significantly elevated among patients with late diagnosis.^6^ Consequently, the molecular mechanism of TGCT progression offers potential biomarkers for clinical treatment, monitoring, and prognosis of TGCT patients.

Circular RNAs are a kind of non-coding RNAs (ncRNAs) with a special circular structure formed through a covalent connection of the 5′ and 3′ ends of linear RNA precursors via a reverse splicing mechanism. CircRNAs are another major family member of ncRNAs following long non-coding RNAs (lncRNAs) and microRNAs (miRNAs). As proved by multiple studies, circRNAs are active in many physiological and pathological processes, and may become biomarkers for tumor treatment and prognosis.^7^ Nucleolar and coiled-body phosphoprotein 1 (NOLC1) is a transcription-like activity protein, important in nuclear assembly and cleavage of precursor rRNA. As a nucleolar GTPase/ATPase, NOLC1 also functions in tumorigenesis and progression processes such as cell cycle, proliferation, migration, and invasion. According to the gene chip, the NOLC1 RNA precursor can equally form exonic circRNA circ-NOLC1 at exon 1–4 via back-splicing. A recent study has shown that circNOLC1 is significantly upregulated in Colorectal Cancer (CRC) and promotes the liver metastasis of CRC by interacting with AZGP1 and Sponging miR-212–5p^8^ Similarly, Chen et al. found that circ-NOLC1 is upregulated in Epithelial Ovarian Cancer (EOC) and has a tumor-promoting effect on EOC cells.^9^ Hence the authors hypothesized that circNOLC1 acts as an oncogene in various tumors, including TGCT. The authors used Gene Expression Profiling Interactive Analysis (GEPIA; www.gepia.cancer-pku.cn) to assess the expression profile of NOLC1 across all tumor samples and paired normal tissues and the highest expression level of NOLC1 was found to be shown in TGCT tumor samples. Therefore, the authors aim to further explore its role in the occurrence and development of TGCT.

Studies on the mechanism of circRNAs in tumorigenesis concentrate mainly on the competitive endogenous RNA hypothesis, that is, circRNAs have miRNAs binding sites, consequently, binding and down-regulating miRNAs function to modulate tumor progression.^10^ The correlation and role of aberrant miRNAs with different types of TGCT has been broadly reported in the past few years^11^; for instance, miR-371a-3p, miRNA-367–3p, miR-371A-3p, miR-372–3p, and miR-373–3p are provided with superior sensitivity and specificity for TGCT diagnosis and can be used as novel biomarkers.^12^, ^13^, ^14^ microRNA-223–3p modulates TGCT cell advancement via targeting FBXW7 to exert a carcinogenic role,^15^ while miR-509–5p represses TGCT cell progression via targeting MDM2 to play an anticancer role.^16^ MiR-140–5p has been confirmed to be critical in various processes of tumors. A study indicated that miR-140–5p inhibits cancer progression via controlling SULT2B1 expression by targeting its 3′-untranslated region in hepatocellular carcinoma.^17^ Additionally, the tumor suppressor role of miR-140–5p in Wilms’ tumor has been reported.^18^ However, how miR-140–5p functions in TGCT progression has not been reported. Bioinformatics approaches have shown that circNOLC1 and miR-140–5p shared binding sites.

Here, the authors investigated the effect of the circNOLC1/miR-140–5p/IGF1R axis in TGCT using bioinformatics analysis and cell functional experiments. The authors supposed that circNOLC1 may mediate the miR-140–5p/IGF1R axis to regulate the biological functions of TGCT cells, thereby affecting the progression of TGCT. CircNOLC1 might be an underlying therapeutic target for TGCT treatment.

## Materials and methods

### Ethical statement and tissue samples

The study was approved by the Ethics Committee of Heji Hospital of Changzhi Medical College, and written informed consent was obtained from all the study participants. Fresh 33 TGCT and 33 regular adjacent testicular tissue samples were collected from patients in the Heji Hospital of Changzhi Medical College. The samples were quickly processed and cryopreserved in liquid nitrogen. All instruments and cryopreservation tubes were treated by autoclaving and immersion in diethylpyrocarbonate solution for specimen retrieval. The tissues were verified via histopathological examination. Inclusion criteria were as follows: 1) Primary surgical patient, 2) Complete records regarding pre-operative chemotherapy and past medical history. Exclusion criteria were as follows: 1) Non-primary surgical patient, 2) Missing or incomplete records regarding pre-operative chemotherapy or past medical history, 3) Previous history of any malignancy.

### Cell culture and transfection

The TCAM-2 and NCCIT cell lines were purchased from the American-type culture collection (VA, USA). The cell morphology was periodically tested, and a short tandem repeat profiling method was used to authenticate the cell lines. The TCAM-2 cells were cultured in Dulbecco's modified eagle medium supplemented with 10 % Fetal Bovine Serum (FBS) (Life Technology Inc.), 1 % antibiotics (100 Units/mL penicillin, and 100 µg/mL streptomycin, Life Technology Inc.), while NCCIT cells were cultured in Roswell Park Memorial Institute 1640 medium supplemented with 10 % FBS (Life Technology Inc.) and 100 U/mL penicillin or streptomycin (Life Technology Inc.).

The cells were seeded in 12-well plates prior to transfection, and complete media without antibiotics was added to each well. When the cells were at a confluence of about 70 %∼90 %, of the cells were transfected with circNOLC1 small interfering RNA (si-circNOLC1) and its corresponding Negative Control (si-NC), circNOLC1 overexpression plasmid (pcDNA3.1-circNOLC1) and its corresponding negative control (pcDNA3.1-NC), miR-140–5p mimic and its corresponding Negative Control (mimic-NC), IGF1R overexpression plasmid (pcDNA3.1-circNOLC1), using Lipofectamine 2000 (11,668–027, Invitrogen, USA) as per the manufacturer's instructions. Si-circNOLC1, miR-140–5p mimic, and their corresponding controls were provided by GenePharma (Shanghai, China). PcDNA3.1-IGF1R, pcDNA3.1-circNOLC1, and pcDNA3.1-NC were synthesized by Genscript (Nanjing, China). The cells were cultured in fresh media after transfection.

### Reverse transcription-quantitative polymerase chain reaction (RT-qPCR)

Total RNA from tissues and cells was extracted using TRIzol (Life Technologies, Carlsbad, CA, USA). According to the manufacturer's instructions, the RNA was reversely transcripted into cDNA by GoldScript one-step RT-PCR Kit (Applied Biosystems, Inc., CA, USA) and the PCR was conducted by SYBR premix Ex Taq™ ⅡPCR Kit (TaKaRa Biotechnology Co., Ltd., Liaoning, China) on the BI7500 PCR amplifier. Primers (Table 1) were designed and synthesized by GenePharma Co., Ltd. (Shanghai, China). U6 was taken as the internal reference of miR-140–5p, and Glyceraldehyde Phosphate Dehydrogenase (GAPDH) was taken as the internal reference of circNOLC1 and IGF1R. The relative expression of miR-140–5p, circNOLC1 and IGF1R were calculated using 2^−ΔΔCt^ method.

**Table 1:** Primer sequence.

**Genes**	**Primer sequence (5′−3′)**
miR-140–5p	Forward: CAGTGGTTTTACCCTATGGTAG
	Reverse: ACCATAGGGTAAAACCACTGTT
U6	Forward: CTCGCTTCGGCAGCACA
	Reverse: AACGCTTCACGAATTTGCGT
circNOLC1	Forward: TGAGCCACCAAAGAACCAGA
	Reverse: AACTTTCGCTCTGGGACCTT
IGF1R	Forward: AACCCCAAGACTGAGGTGTG
	Reverse: TGACATCTCTCCGCTTCCTT
GAPDH	Forward: AGAAGGCTGGGGCTCATTTG
	Reverse: AGGGGCCATCCACAGTCTTC

### RNase R treatment

The TCAM-2 and NCCIT cells were treated with RNase R (2 U/µg, GeneSeed, Guangzhou, China) and incubated after inactivation. The treated RNAse was reversely transcribed using divergent or convergent primers and then examined. The quantities of circNOLC1 and NOLC1were analyzed with real-time quantitative Reverse Transcription Polymerase Chain Reaction (qRT-PCR).

### Western blot analysis

Total protein was extracted from tissues and cells after transfection according to the instructions of the protein extraction kit (Thermo, USA). Protein concentration was determined using a bicinchoninic acid kit (Beyotime Biotechnology, Shanghai, China). The extracted proteins (30 µg) were added to the loading buffer, and proteins were separated using 10 % polyacrylamide gel electrophoresis and transferred to a polyvinylidene fluoride membrane. The membrane was then blocked using 5 % bovine serum albumin and later incubated with primary antibody anti-IGF1R (CD221, ThermoFisher, MA, USA) (1:1000) and anti-GAPDH (1:3000) (Abcam, Cambridge, MA, USA) at 4 °C overnight. The samples were then incubated with the corresponding secondary antibodies (Shanghai Miaotong Biotechnology Co., Ltd., Xuhui District, Shanghai, China) at 37 °C for 2h The protein band development was visualized using a chemiluminescence reagent, and GAPDH was used as an internal reference. The Bio-rad Gel Dol EZ imager (Gel DOC EZ IMAGER, Bio-rad, California, USA) was used to check the developed bands and analyzed through Image J software. The experiment was repeated three times.

### 3-(4, 5-dimethylthiazol-2-yl)-2, 5-diphenyltetrazolium bromide (MTT) assay

The cells were collected, homogenized, and seeded in 96-well plate, at a density of 8 × 10^3^ cells per well. Next, 200 μL of culture media was added and incubated for 24 h, 48 h, and 72h Next, 5 g/L of MTT reagent (20 μL) was added to each well. The cells were then grown for 4 h, followed by the addition of DMSO (150 µL) after the removal of MTT. Optical cell Density (OD) was finally determined in a microplate reader at 490 nm using a microplate reader (Bio-tekEL×800, US). The experiment was carried out in triplicates.

### Colony formation assay

Approximately 3 × 10^2^ cells/well of TCAM-2 or NCCIT cells were plated in triplicate in a 6-well plate. The cells were cultured for 9 days, and cell colonies were later fixed in methanol and stained in crystal violet (0.1 %) for 1 h at room temperature. Later, the plates were submerged in a water bath for 1 hour to achieve a complete wash. Finally, the clones were counted and photographed.^19^ The assay was done in triplicates.

### Flow cytometry

The cells were washed in cold PBS, resuspended in sterile PBS, and stained with 5 μL Annexin V-FITC and 5 μL Propidium Iodide (PI) (BD Biosciences) at 25 °C for 15 min in the dark. The apoptosis rate was determined by Flow cytometry (FACSCanto™) and was eventually analyzed by CellQuest Pro (BD Biosciences). The assay was done in triplicates.

### Transwell assay

Transwell assay was used to determine cell invasion, as previously described.^20^ A serum-free matrix mixture was prepared by taking 80 μL of serum-free medium pre-cooled at 4 °C and adding 20 μL of Matrigel matrix gel (BD Biosciences, USA). The mixture was uniformly added to the upper chamber of a Transwell Chamber (Corning Corporation, Corning, NY, USA) and placed in a 5 % CO_2_ incubator at 37 °C for 6 h until the matrix gel solidified. Logarithmically growing cells (1 × 10^5^) were diluted in 200 μL of serum-free DMEM medium and inoculated into both the upper chamber with matrix gel and without matrix gel. The lower chamber contained 800 μL of medium with 10 % fetal bovine serum. The chambers were then incubated in an incubator at 37 °C and 5 % CO_2_ for 36 h. After removing the cells and washing them three times with PBS, they were fixed with a solution containing 4 % paraformaldehyde and stained using a solution containing 1 % crystal violet. Following another wash with PBS, any remaining cells on the surface were wiped off using a cotton swab. After air drying naturally, the cells were photographed under an optical microscope and counted. Nine random fields of view were counted to calculate an average value. The assays were done in triplicate.

### The luciferase activity assay

According to the bioinformatics website Targetscan (http://www.targetscan.org), the sequence of circNOLC1 and 3′-UTR of IGF1R containing the potential binding sites of miR-140–5p were synthesized and purchased from RiboBio (Guangzhou, China). Then the fragments of circNOLC1 or IGF1R 3′UTR possessing the Wild-Type (WT) and Mutant (MUT) binding site of miR-140–5p were inserted into the psi-CHECK2 fluorescent reporter gene plasmid (Promega Company, USA) to generate WT-circNOLC1, MUT-circNOLC1, WT-IGF1R-3′UTR, and MUT-IGF1R. A luciferase reporter assay was used to validate the prediction.^21^ Wild-type and mutant circNOLC1 or IGF1R plasmid and miR-140–5p mimic and its negative control were separately transfected into the cells using LipofectamineTM 2000 transfection reagent following the manufacturer's instructions. The cells were harvested 48 h later, and the luciferase activity was assessed through the Dual-Luciferase Reporter Assay System (Promega). All the assays were done in triplicates.

### RNA-pull down assay

Briefly,^22^ Biotin-labeled miR-140–5p wild-type plasmid and biotin-labeled miR-140–5p mutant plasmid (50 nM each) were separately transfected into cells. After 48 h of transfection, the cells were collected and washed with PBS, followed by incubation with specific cell lysates (Ambion, Austin, Texas, USA) for 10 min. Subsequently, 50 mL of the sample cell lysate was divided. The remaining lysate was then incubated with M-280 streptavidin magnetic beads (purchased from Sigma, St. Louis, MO, USA) pre-coated with RNase-free yeast tRNA. Incubation was carried out at 4 °C for 3 h followed by washing twice with cold cracking solution, three times with low salt buffer, and once with high salt buffer. An antagonistic miR-140–5p probe was used as a negative control. Total RNA was extracted using Trizol and the expression level of circNOLC1 was determined by qRT-PCR.

### Statistical analysis

GraphPad Prism 8 was used for data analysis. The data were presented as mean ± Standard Deviation (SD). The comparison between the two groups was via *t*-test. One-way analysis of variance (ANOVA) was used for the comparison among multiple groups, and Tukey's multiple comparisons test for pairwise comparisons after ANOVA analysis. Pearson test was used for correlation analysis; *p* < 0.05 was considered statistically significant.

## Results

### CircNOLC1 is expressed at high levels in testicular germ cell tumor

**Fig. 1:**
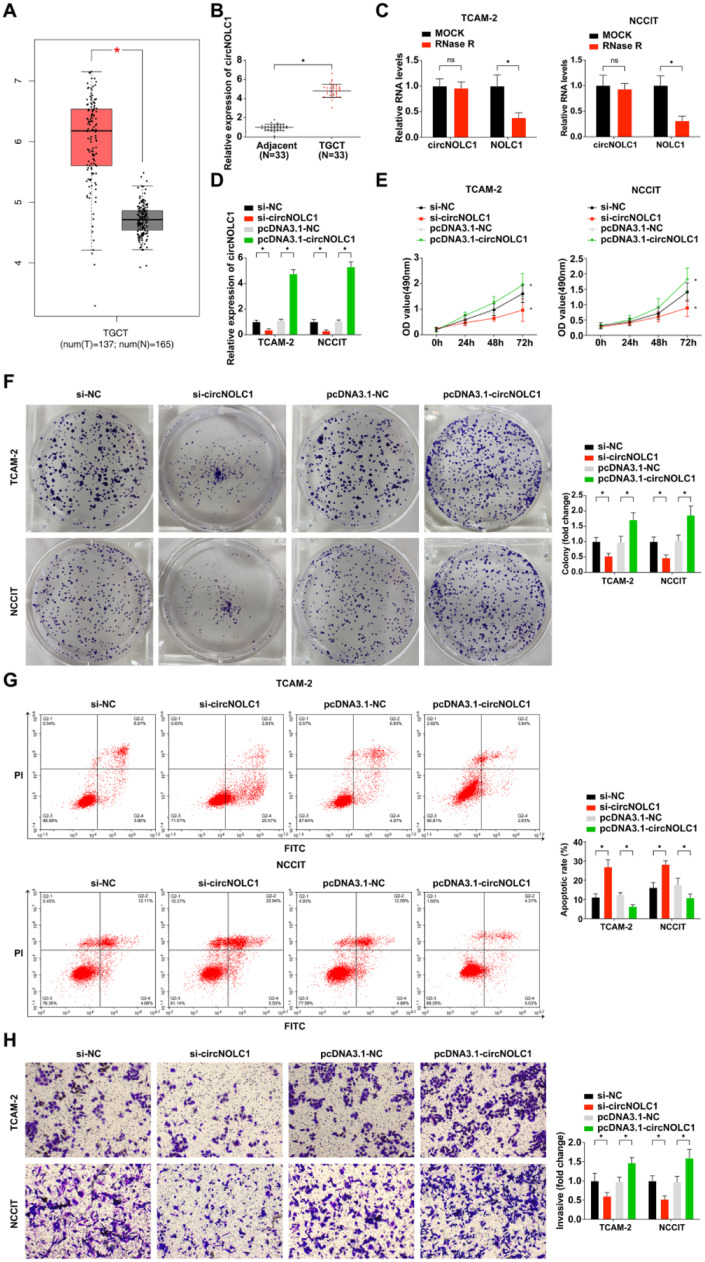
**CircNOLC1 is expressed at high levels in testicular germ cell tumor.** (A) CircNOLC1 expression in TGCT tissues compared with normal tissues using the GEPIA Database; (B) Examination of circNOLC1 in TGCT and para-cancer tissues using RT-qPCR; (C) Determination of the circular characteristics of circNOLC1 through RNase R treatment; (D) Examination of circNOLC1 in TGCT cells done through RT-qPCR; (E) Detection of TGCT cell advancement was via MTT assay. (F) Test of clone formation of TGCT cells was via plate cloning assay; (G) Examination of TGCT cell progression using FCM; (H) Detection of TGCT cell development was via Transwell assay. *p < 0.05, the data were presented as mean ± SD.

CircNOLC1 expression was investigated in TGCT compared with normal controls following the instruction of the GEPIA website creator. The expression of CircNOLC1 was obviously upregulated in TGCT (Fig. 1A). Then the authors detected the expression of circNOLC1 in 33 TGCT tissues and their adjacent normal tissues by qRT-PCR, and the present result further verified the result of GEPIA website analysis (Fig. 1B).

Furthermore, TGCT patients were divided into two groups according to the median value of the relative expression level of CircNOLC1: low expression group and high expression group, and the relationship between circNOLC1 expression and the clinicopathological characteristics of TGCT patients was analyzed. As shown in Table 2, the pathological grade, clinical stage, and lymph node metastasis of TGCT patients were significantly correlated with circNOLC1 expression.

**Table 2:** Association of circNOLC1 with pathological parameters of TGCT.

**Clinical indexes**	***n*=33**	**circNOLC1**		**p**
		**The declined (17)**	The elevated (16)	
Age(years)				0.353
≥ 35	13	8	5	
< 35	20	9	11	
Tumor diameter (cm)				0.611
< 2	18	10	8	
≥ 2	15	7	8	
TNM stage				0.006
Tis‒T1	12	10	2	
T2‒T4	21	7	14	
Differentiation				0.008
Well	14	11	3	
Poor	19	6	13	
Lymph node metastasis				0.024
Yes	19	13	6	
No	14	4	10	

The authors also found that the linear mRNA NOLC1 was digested after RNase R treatment, while circNOLC1 was not (Fig. 1C), implying that circNOLC1 was a circular RNA. Next, in order to further explore the biological effects of circNOLC1 on TGCT, the authors transfected pcDNA3.1-circNOLC1, si-circNOLC1or their negative controls into TGCT cells, and the transfection efficiency was verified by qRT-PCR. It was found that si-circNOLC1 effectively knocked down the expression of circNOLC1 in TGCT cells while pcDNA3.1-circNOLC1 successfully upregulated the expression of circNOLC1 in TGCT cells (Fig. 1D). The results of MTT, colony-forming assay, and flow cytometry showed that si-circNOLC1 could restrain the proliferation and clonogenesis of TGCT cells and promote the apoptosis of TGCT cells, whereas pcDNA3.1-circNOLC1 had the opposite effect, as shown in Fig. 1E‒G. Moreover, the transwell assay result revealed that knocking down circNOLC1 inhibited the invasion of TGCT cells and up-regulation of circNOLC1 promoted the invasion of TGCT cells (Fig. 1H).

### CircNOLC1 acts as an miR-140–5p sponge in TGCT

**Fig. 2:**
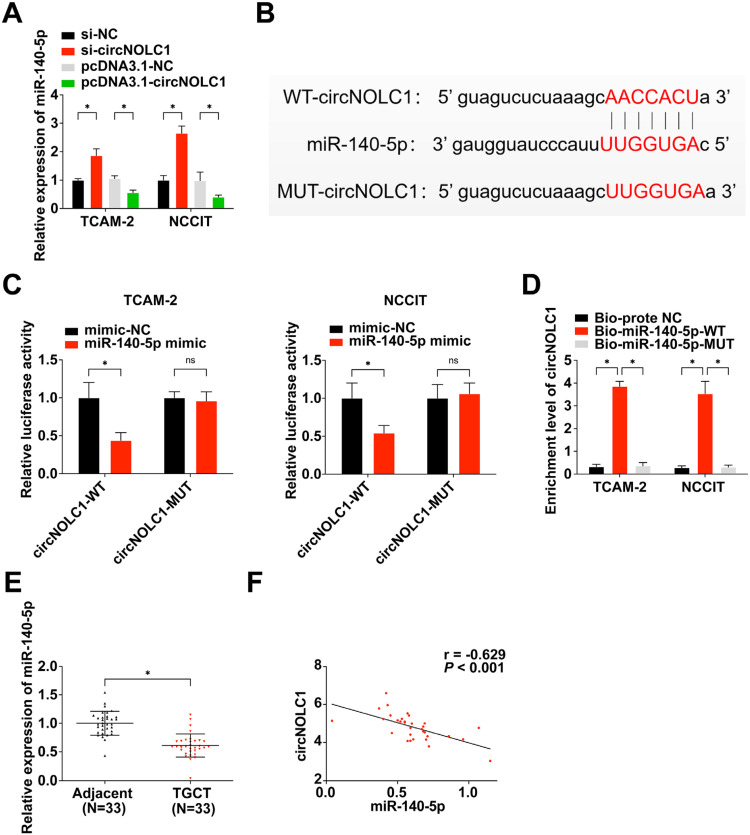
**CircNOLC1 acts as an miR-140–5p sponge in TGCT.** (A) Detection of miR-140–5p in TGCT cells using RT-qPCR; (B) Prediction of the binding site of circNOLC1 and miR-140–5p; (C) Verification of the combination of circNOLC1 and miR-140–5p done using dual-luciferase; (D) Test of enrichment of miR-140–5p to circNOLC1 through RNA pull-down; (E) RT-qPCR for examination of miR-140–5p in TGCT and para-cancer tissues; (F) MiR-140–5p was correlated with circNOLC1. *p < 0.05, the data were presented as mean ± SD.

QRT-PCR analysis revealed that the overexpression of circNOLC1 resulted in the inhibition of miR-140–5p expression, while knockdown of circNOLC1 led to an up-regulation of miR-140–5p expression (Fig. 2A). Bioinformatics analysis indicated a potential binding between circNOLC1 and miR-140–5p (Fig. 2B), which was further confirmed by a dual luciferase reporter assay (Fig. 2C). The results showed that the luciferase activity of the circNOLC1-WT group was significantly decreased in the miR-140–5p mimic group compared with the mimic-NC group in cells. No significant difference in luciferase activity was observed between the miR-140–5p mimic and mimic-NC group in cells transfected with the circNOLC1-MUT vector. In addition, RNA pull-down experiment demonstrated that the enrichment level of circNOLC1 was significantly increased in the Bio-miR-140-WT group compared to the Bio-probe negative control group; however, there was no significant difference in enrichment level observed in the Bio-miR-140-MUT group (Fig. 2D). These findings collectively support that circNOLC1 acts as a competing endogenous RNA for miR-140–5p and regulates its expression. Furthermore, the authors examined the expression level of miR-140–5p in TGCT tissues and analyzed its correlation with circNOLC1. The authors found that miR-140–5p was reduced in TGCT tissues which was negatively correlated with circNOLC1 (Fig. 2E‒F).

### MiR-140–5p reverses the carcinogenic effects of circNOLC1 on TGCT

In order to further investigate the mechanism of circNOLC1/miR-140–5p in the malignant phenotype of TGCT, the authors designed a rescue experiment.

**Fig. 3:**
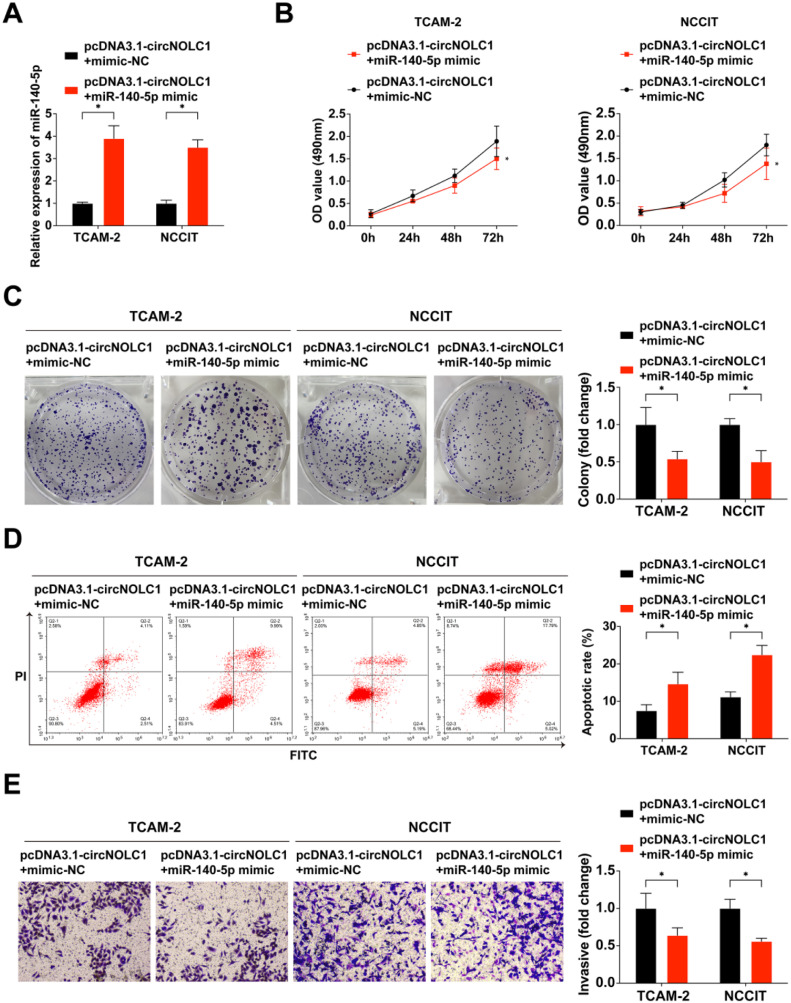
**MiR-140–5p reverses the carcinogenic effects of circNOLC1 on TGCT.** (A) Detection of miR-140–5p in TGCT cells was via RT-qPCR; (B) Examination of TGCT cell progression was via MTT assay; (C) Test of clone formation of TGCT cells was via plate cloning assay; (D) Examination of TGCT cell progression was via FCM; (E) Test of the TGCT cell advancement was via Transwell assay. *p < 0.05, the data were presented as mean ± SD.

The TCAM-2 and NCCIT cells were co-transfected with pcDNA3.1-circNOLC1+mimic-NC or pcDNA3.1-circNOLC+miR-140–5p mimic. The data of qRT-PCR analysis validated successful upregulation of miR-140–5p expression by miR-140–5p mimic (Fig. 3A). Cell function assays revealed that the miR-140–5p mimic effectively reversed pcDNA3.1-circNOLC-mediated promotion of cell proliferation and invasion while also reversing its inhibitory effect on apoptosis, as illustrated in Fig. 3B‒E. These findings suggest that circNOLC1 promotes the malignant behavior of TGCT progression through sponging miR-140–5p

### IGF1R is the target gene of miR-140–5p

**Fig. 4:**
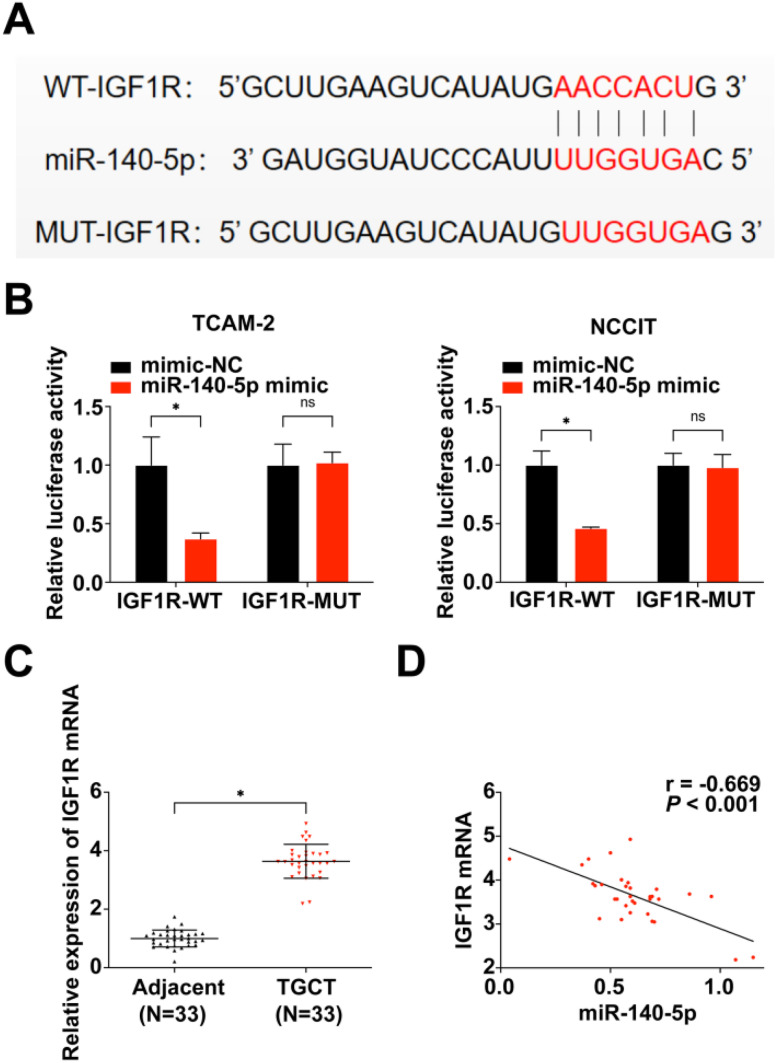
**IGF1R is the target gene of miR-140–5p** (A) Prediction of the binding site of IGF1R and miR-140–5p; (B) Verification of the targeting of IGF1R with miR-140–5p done using dual-luciferase; (C) RT-qPCR for the detection of IGF1R in TGCT and para-cancer tissues; (D) MiR-140–5p was associated with IGF1R mRNA. *p < 0.05, the data were presented as mean ± SD.

Next, the authors proceeded with the investigation of miR-140–5p's mechanism and biological function in TGCT cell development. The binding site of IGF1R gene to miR-140–5p was predicted by bioinformatics software (Fig. 4A). For verification, the authors designed mutant sequences deleting miR-140–5p binding sites within IGF1R 3′UTR as well as wild-type sequence insertion reporter plasmids. Subsequently, cells were co-transfected with miR-140–5p mimic and either wild-type or mutant recombinant plasmids for luciferase activity detection. The results demonstrated that while there was no significant effect on luciferase activity intensity in the mutant group upon transfection with miR-140–5p mimic, a notable reduction was observed in luciferase activity intensity within the wild-type reporter group (Fig. 4B). Furthermore, analysis of IGF1R mRNA expression levels in TGCT tissues along with their correlation to miR-140–5p expression revealed an up-regulation of IGF1R mRNA in TGCT tissues which exhibited a negative correlation with miR-140–5p expression (Fig. 4C‒D). Summarily, these observations confirm that IGF1R is the target gene of miR-140–5p

### IGF1R is a functionally important target of miR-140–5p in modulating TGCT cell functional behaviors

**Fig. 5:**
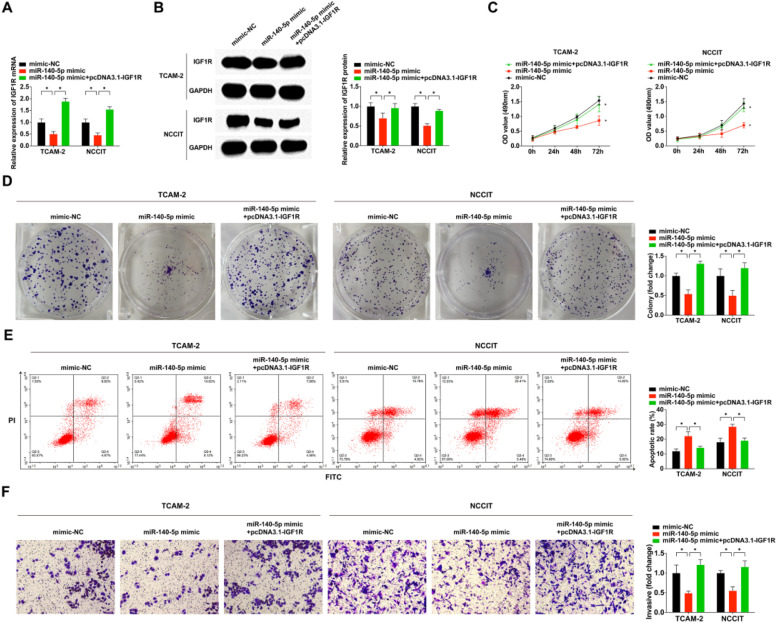
**IGF1R is a functionally important target of miR-140–5p in modulating TGCT cell functional behaviors.** (A) RT-qPCR for detection of IGF1R in TGCT cells; (B) A western blotting assay to determine IGF1R expression in TGCT cells; (C) MTT assay for the assessment of TGCT cells proliferation; (D) Assessment of clone formation of TGCT cells through plate cloning assay; (E) Test of TGCT cell progression was via FCM; (F) Examination of TGCT cell advancement was done via Transwell assay. * *p* < 0.05, the data were presented as mean ± SD.

To further elucidate the underlying mechanism of miR-140–5p/IGF1R in the malignant phenotype of TGCT, the authors separately transfected mimic-NC, miR-140–5p mimic, and co-transfected miR-140–5p mimic with pcDNA3.1-IGF1R into cells. Subsequent qRT-PCR and WB experiments demonstrated that the miR-140–5p mimic effectively suppressed IGF1R mRNA and protein expression levels, but pcDNA3.1-IGF1R successfully reversed the inhibitory effect exerted by miR-140–5p mimic on IGF1R expression (Fig. 5A‒B). The results obtained from MTT assay, colony-forming assay, and flow cytometry analyses revealed that overexpression of miR-140–5p significantly impeded TGCT cell proliferation and invasion while promoting apoptosis (Fig. 5C‒F). Notably, the rescue experiment results showed that pcDNA3.1-IGF1R counteracted these antitumor effects mediated by miR-140–5p in TGCT cells. Collectively, these findings strongly suggest that through its regulation of IGFIR, miR-140–5p plays a crucial role in suppressing TGCT cell development.

### CircNOLC1 acts as a miRNA sponge of miR-140–5p to regulate the expression of igf1r

**Fig. 6:**
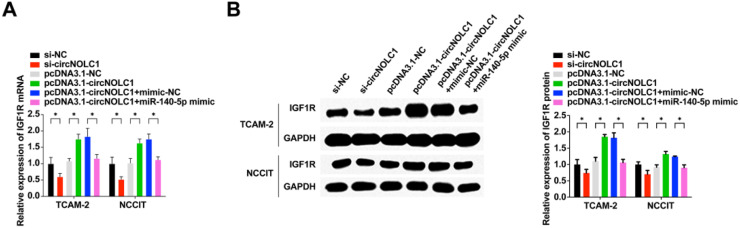
**CircNOLC1 acts as an miRNA sponge of miR-140–5p to regulate the expression of IGF1R.** (A) Test of IGF1R mRNA expression in TGCT cells was via RT-qPCR; (B) Examination of IGF1R protein expression in TGCT cells was via WB. * *p* < 0.05, the data were presented as mean ± SD.

Having demonstrated the function of circNOLC1 as a sponge of miR-140–5p and IGF1R as a direct target gene of miR-140–5p, the authors wanted to explore whether circNOLC1 could modulate IGF1R expression by acting as a competing endogenous RNA (ceRNA) for miR-140–5p The expression of IGF1R mRNA and protein in cells was detected by qRT-PCR and WB (Fig. 6). It was found that IGF1R expression was down-regulated after circNOLC1 knockdown or miR-140–5p up-regulation. On the other hand, IGF1R expression was upregulated after circNOLC1 was upregulated, and miR-140–5p mimic could partially reverse the promoting effect of circNOLC1 on IGF1R expression. Hence suggested that circNOLC1 can regulate the expression of IGF1R, and this regulatory effect is achieved by sponging miR-140–5p It was further confirmed that circNOLC1 may promote the occurrence and development of TGCT by acting as a ceRNA for miR-140–5p to induce IGF1R expression.

## Discussion

CircRNAs are new endogenous RNAs critical in many epigenetic modulations of pathological and physiological conditions. Studies have confirmed that circRNAs can also be aberrantly expressed in human testicular tissue-related diseases in the past few years, thus leading to disease advancement. Further studies have reported circRNA-9119 as an endogenous competitive RNA, which binds to miR-136 and miR-26a, subsequently leading to the regulation of testicular inflammation.^23^ According to Mo-qi and the team, Hsa_circ_0000116 is critical in modulating spermatogenesis, hence a potential biomarker for non-obstructive azoospermia diagnosis and treatment.^24^ However, the role of circNOLC1 in TGCT has not been clarified.NOLC1 is the host gene of circNOLC1, initially detected as a nuclear localization signal binding, which acts as a chaperone that shuttles the nucleolus with the cytoplasm.^25^^,^^26^ Previous studies clarified that NOLC1 plays various roles in different cancers. For instance, Yuan F et al. observed that NOLC1 is reduced in human hepatocellular carcinoma tissues.^27^ Contrarily, Hwang and colleagues reported that NOLC1 induces tumorigenesis in nasopharyngeal carcinoma.^28^ In the present study, combined the data with the data from GEPIA analysis, the authors found circNOLC1 was significantly elevated in TGCT tissues. And the high expression of circNOLC1 was directly correlated with the advanced clinical stage, pathological grade, and lymph node metastasis of TGCT patients. Furthermore, the authors investigated the effect of circNOLC1 on TGCT in vitro. The results demonstrated that circNOLC1 knock-down suppressed TGCT cell progression, whereas circNOLC1 overexpression induced the progression of TGCT cells. In agreement with the present findings, Chen and colleagues confirmed that circ-NOLC1 accelerates epithelial ovarian cancer occurrence and progression, while Chen W et al. maintain that circ-NOLC1 induces prostate cancer progression via miR-647/PAQR4 axis.^28^^,^^29^

It's well known that circRNAs could function as miRNA molecular “sponges”, thus impacting cancer progression.^30^^,^^31^ In further exploring the molecular mechanism of circNOLC1 in TGCT progression, the authors found that circNOLC1 binds and downregulates miR-140–5p through bioinformatic analysis and mechanism experiments. Moreover, the authors performed rescue experiments that indicated miR-140–5p could rescue the pro-tumor role of circNOLC1 in TGCT. These findings agree with accumulating evidence that confirmed the anti-tumor role of miR-140–5p in various cancers, such as pancreatic cancer,^32^ gastric cancer,^33^ osteosarcoma,^34^ and myeloma.^35^

IGF1R is a transmembrane receptor of the tyrosine kinase family.^36^ According to previous reports, aberrant IGF1R expression induces the initiation and progression of multiple cancers.^37^, ^38^, ^39^ Particularly, IGF1R expression is elevated in TGCT compared to the normal tissues.^40^ The authors also reported an increased IGF1R expression in TGCT tissues and it was negatively correlated with miR-140–5p expression. Previous studies have revealed that IGF1R is involved in multiple cancers via being targeted by some miRNAs,^41^^,^^42^ including miR-140–5p^43^

Herein, the present work, confirmed IGF1R is targeted by miR-140–5p and the authors verified IGF1R is a functionally important target of miR-140–5p in modulating TGCT cell malignant phenotype via rescue experiments. Finally, through qRT-PCR and WB the authors found that IGF1R expression was promoted by circNOLC1 overexpression, and miR-140–5p mimic could partially reverse the promoting effect of circNOLC1 on IGF1R expression. Thereby confirms the initial assumption that circNOLC1 mediates miR-140–5p/IGF1R axis to regulate the biological functions of TGCT cells.

As the authors first confirmed that circNOLC1/miR-140–5p/IGF1R axis promotes TGCT proliferation and invasion in vitro, this work may reveal a novel mechanism involved in TGCT progression, as well as provide a new perspective for the early diagnosis and targeted therapy of TGCT. But the authors have to admit there are still deficiencies in this study. First, the authors didn't perform in vivo experiment due to lack of funding. It will be the next research plan. Second, even though the authors revealed that circNOLC1 was abnormally upregulated in TGCT tissues and was directly correlated with the advanced clinical stage, pathological grade, and lymph node metastasis of TGCT patients, the sample size of patients is very limited and the expression of circNOLC1 should be further evaluated in blood, urine, and exosomes to improve its clinical application value. Last, the authors only explored the ceRNA mechanism of circNOLC1 in TGCT, which does not mean that there are no other important regulatory networks, and the mechanisms in other fields need to be further explored.

## Conclusion

In conclusion, the authors have found that circNOLC1 could function as a ceRNA to sponge miR-140–5p, thereby regulating the malignant behaviors of TGCT cells via targeting IGF1R. This study may be helpful for TGCT treatment, while further efforts are still needed.

## Authors’ contributions

Feng Lin designed the research study. Xianming Yao and ShuoShuo Zhang performed the research. Feng Lin and Huajun Yang provided help and advice on the experiments. Xianming Yao, ShuoShuo Zhang and Huajun Yang analyzed the data. Feng Lin wrote the manuscript. Huajun Yang reviewed and edited the manuscript. All authors contributed to editorial changes in the manuscript. All authors read and approved the final manuscript.

## Ethics approval

The present study was approved by the Ethics Committee of Heji Hospital of Changzhi Medical College (n° 202010SX75) and written informed consent was provided by all patients prior to the study start. All procedures were performed in accordance with the ethical standards of the Institutional Review Board and The Declaration of Helsinki, and its later amendments or comparable ethical standards.

## Funding

Not applicable.

## Availability of data and materials

The datasets used and/or analyzed during the present study are available from the corresponding author upon reasonable request.

## Conflicts of interest

The authors declare no conflicts of interest.
